# The arginine sensing and transport binding sites are distinct in the human pathogen *Leishmania*

**DOI:** 10.1371/journal.pntd.0007304

**Published:** 2019-04-24

**Authors:** Harsh Pawar, Madhu Puri, Renana Fischer Weinberger, Rentala Madhubala, Dan Zilberstein

**Affiliations:** 1 Faculty of Biology, Technion-Israel Institute of Technology, Haifa, Israel; 2 School of Life Sciences, Jawaharlal Nehru University, New Delhi, India; Bernhard Nocht Institute for Tropical Medicine, Hamburg, Germany, GERMANY

## Abstract

The intracellular protozoan parasite *Leishmania donovani* causes human visceral leishmaniasis. Intracellular *L*. *donovani* that proliferate inside macrophage phagolysosomes compete with the host for arginine, creating a situation that endangers parasite survival. Parasites have a sensor that upon arginine deficiency activates an Arginine Deprivation Response (ADR). *L*. *donovani* transport arginine via a high-affinity transporter (LdAAP3) that is rapidly up-regulated by ADR in intracellular amastigotes. To date, the sensor and its ligand have not been identified. Here, we show that the conserved amidino group at the distal cap of the arginine side chain is the ligand that activates ADR, in both promastigotes and intracellular amastigotes, and that arginine sensing and transport binding sites are distinct in *L*. *donovani*. Finally, upon addition of arginine and analogues to deprived cells, the amidino ligand activates rapid degradation of LdAAP3. This study provides the first identification of an intra-molecular ligand of a sensor that acts during infection.

## Author summary

*Leishmania donovani*, the causative agent of visceral leishmaniasis, leads a digenetic life cycle as a flagellated promastigote in the vector sandfly and aflagellated amastigote within phagolysosomes of infected macrophages. Arginine is an essential amino acid for *Leishmania* which possesses a high specificity arginine transporter (LdAAP3), a protein that imports the amino acid into parasite cells. Arginine is primarily utilized in de novo protein synthesis and for biosynthesis of trypanothione via the polyamine pathway. It was previously reported by our group that *L*. *donovani* senses lack of arginine in the surrounding micro environment and activates a unique arginine deprivation response (ADR) pathway, thus upregulating the expression of LdAAP3 as well as other transporters. In the present study, we identified the region on the arginine molecule which is the ligand that activates ADR. We show that the conserved amidino group at the distal cap of the arginine side chain is the ligand that activates/suppresses ADR. Using arginine analogues that contain this group we observed that arginine sensing and transport are distinct in *L*. *donovani*, both in axenic promastigotes and intracellular amastigotes. Additionally, the arginine sensor responds to both arginine starvation and sufficiency.

## Introduction

*Leishmania donovani* is a parasitic protozoan that causes visceral leishmaniasis (kala-azar) in humans that is almost always fatal [1]. This organism is an obligatory intracellular pathogen which cycles between the acidic phagolysosome of macrophages (intracellular amastigote) and the relatively alkaline mid-gut of female sand flies (extracellular promastigote) [2]. Shifting between the two distinct environments requires flexible adaptation mechanisms, and the ability to sense and rapidly respond to fluctuations in vector and host environments [3]. We use *L*. *donovani* as a model organism to investigate the molecular tools parasites have developed to overcome the harsh environments they encounter in the host and vector.

Arginine is a semi-essential amino acid for mammals and is required for protein synthesis and other metabolic pathways like the synthesis of urea, polyamines and creatinine [4]. On the other hand, arginine is an essential amino acid for *Leishmania*, as the parasites cannot endogenously synthesize it and rely on its uptake from host arginine pools. Additionally, arginine is the sole precursor for the production of spermidine, and subsequently trypanothione, in *Leishmania* [5,6]. The parasite utilizes arginine for polyamine biosynthesis as it lacks both catalases and classical seleno-containing glutathione peroxidases and thus has to rely on trypanothione for maintaining redox balance [5]. Arginine is transported within *L*. *donovani* via a high-affinity arginine transporter (LdAAP3), which is specifically localized to the membrane of the flagellum and glycosomes [7]. LdAAP3 is a highly specific transporter and only a few compounds affect/inhibit it [8]. When over-expressed, LdAAP3 also localizes on the plasma membrane [8].

During infection with *L*. *donovani*, macrophages employ defense mechanisms such as nitric oxide (NO) and reactive oxygen species (ROS) production [9]. The synthesis of NO by macrophages requires arginine. The intracellular parasites increase arginase I activity in macrophages, which restricts the amount of arginine available for NO production, and also increase the production of trypanothione, thereby neutralizing ROS and facilitating a safe niche for themselves inside the macrophages [10,11]. Therefore, *Leishmania* is locked in competition with the host for arginine. *L*. *donovani* modulates infected host macrophages to stimulate arginine transport by up-regulating the expression of the cationic amino acid transporter 2- CAT2B (*SLC7A2*), thereby ensuring its own survival [12].

We have previously reported the presence of an arginine deprivation response (ADR) in *L*. *donovani*, which monitors arginine levels in its environment. Genome-scale transcriptomics identified six transporters that up-regulate upon ADR activation in *L*. *donovani*, which include, in addition to LdAAP3, the pteridine, folate and three putative transporters [7]. Interestingly, we observed that ADR activation occurs in intracellular amastigotes, thereby supporting the notion that parasites undergo arginine deprivation during development in the phagolysosome.

Most eukaryote amino acid sensors respond to sufficiency or deficiency by activating a mTOR signaling cascade [13]. In contrast, we have previously shown that the *L*. *donovani* arginine sensor responds to environmental arginine deprivation by activating a mitogen-activated protein kinase 2-mediated signaling pathway that within minutes up-regulates the expression of the ADR genes [7]. This arginine sensor was identified for the first time and was reported to induce a response in the absence of its ligand. Furthermore, the addition of arginine to ADR-activated promastigotes induced a rapid degradation of the *LdAAP3* protein to its homeostatic level. This indicated that the arginine sensor activates two distinct pathways: the ADR and an arginine sufficiency response. To date, we have not identified the *L*. *donovani* arginine sensor or its ligand.

This study identifies the regions on the arginine molecule which are essential for ADR activation and binding to the arginine transporter. We show that the conserved amidino group at the distal cap of the arginine side chain is the ligand that activates/suppresses ADR, both in axenic promastigotes and intracellular amastigotes. Using arginine analogues and additional compounds that contain this group, but lack the α amino group, we observed that arginine sensing and transport binding sites are distinct in *L*. *donovani*. Furthermore, these analogues affect arginine sensing of intra-lysosomal amastigotes. Sensor specificity is very high as any modification of the amidino group results in non-recognition of the arginine analogue by the sensor and thus has no effect on ADR. This study comprises the first in-depth analysis of arginine sensing in *Leishmania*.

## Materials and methods

### Chemicals

L-arginine, D-arginine, Pentamidine, Canavanine, N-Methyl L-arginine acetate (NMLAA), Nω-Nitro-L-arginine methyl ester (L-NAME), Nω-Nitro-L-arginine (L-NNA), L-citrulline, 3-Ureidopropionic acid and 4-{[5-(4-aminophenoxy)pentyl]oxy}phenylamine were obtained from Sigma-Aldrich, USA. All other materials used in this study were of analytical grade and commercially available.

### *Leishmania* cell culture

Promastigote cultures of the *L*. *donovani Bob* strain (*Ld*Bob strain/MHOM/SD/62/1SCL2D), initially obtained from Dr Stephen Beverley (Washington University, St. Louis, MO, USA), and *L*. *donovani* 1S strain, (MHOM/SD/00/1S) were used in this study. Promastigotes were cultured at 26°C in M199 medium (Sigma-Aldrich, USA), supplemented with 100 units/ml penicillin (Sigma-Aldrich, USA), 100 μg/ml streptomycin (Sigma-Aldrich, USA) and 10% heat-inactivated fetal bovine serum (FBS; Biowest).

### THP-1 cell culture and infection

THP-1 cells, an acute monocytic leukaemia-derived human cell line (ATCC, TIB-202TM) were used for all experiments. They were cultured in RPMI-1640 (Sigma-Aldrich, USA) medium supplemented with 10% heat-inactivated FBS, 100 units/ml penicillin and 100 μg/ml streptomycin at 37°C in a humidified atmosphere. For infection assays, 0.5 x 10^6^ cells/ml were seeded in RPMI-1640 medium containing 10% FBS. The cells were treated with 50 ng/ml of phorbol 12-myristate 13-acetate (PMA; Sigma-Aldrich, USA) for 48 h to induce their differentiation into macrophage-like cells. Immediately before infection, the cells were washed once with phosphate buffered saline (PBS) and incubated in RPMI medium (Sigma-Aldrich, USA) containing 0.1 mM arginine (unless stated otherwise), and supplemented with 10% heat-inactivated FBS, 100 units/ml penicillin, and 100 μg/ml streptomycin. Promastigotes in the late log-phase were added to cells at a ratio of 20:1 and incubated at 37°C in a humidified atmosphere for 5 h. Extracellular parasites were removed by washing the cells five times with PBS. Thereafter, the cells were incubated in RPMI medium containing 0.1 mM arginine (unless stated otherwise) at 37°C in a humidified atmosphere for 2 h, 24 h, or 48 h.

### Arginine deprivation

Mid-log phase promastigotes (1×10^7^ cells/ml) were used for arginine deprivation studies. The cells were washed with Earl’s salt solution twice and re-suspended in arginine deficient Medium 199 (Biological Industries Ltd.). Arginine deprivation was carried out at 26°C for specified time periods and was concluded by transferring the cells to ice. Arginine deprived cells were washed twice with Earl’s salt solution before being used for transport assays, Northern and Western blot analysis.

### Macrophage phagosome isolation

THP-1 cells were cultured in DMEM medium with 10% FBS. Magnetic beads of 3 μm size were added to flasks containing THP-1 cells. The isolation of intact macrophage phagosomes was carried out as described by Kuhnel *et al*. [14].

### Phagosome amino acid content analysis

The isolated phagosomes were then resuspended in 1 ml of ice-cold phosphate-buffered saline plus 1 ml of 5 N perchloric acid, vortexed, and incubated on ice for an additional 10 min. Perchloric acid lysates were centrifuged in a microcentrifuge at 14,000 rpm for 10 min at 4 °C, and 232 μl of 5 N KOH was added to the supernatant to titrate the sample pH to 7.0. Additional centrifugation was performed under the aforementioned conditions, and 200 μl aliquots were analyzed for amino acid content by the method described by Fekkes *et al*. [15]. The analyses were carried out at the Medical Biochemistry Laboratory at Rambam Medical Center in Haifa.

### Transport assays

Uptake of 25 μM [^3^H]arginine (600 mCi/ mmol), by mid-log phase parasites was determined by the rapid filtration technique of Mazareb et al. as reported [16]. To determine initial rates of transport, transport measurements were performed on 1 x 10^8^ promastigotes exposed to radiolabel for up to 2 min. The amount of radiolabel associated with the cells was linear with time over the 2-min time course of the transport assay.

### Northern blot analyses

Total RNA from *L*. *donovani* promastigotes (either deprived for arginine or non-deprived) was prepared using the Tri-reagent protocol and subjected to Northern blotting for *LdAAP3* as described before [17]. Probes were amplified using the following primers: LinJ.31.900 *LdAAP3* Forward: 5'-GCTGTGACGGGGTCAGTG-3' and. Reverse: 5'-GTACGTCGCCAGCCAGTG-3'. LdBPK_101450.1 pteridine transporter Forward: 5’- ATGACCGTTGGTCAGCAGA-3’ and Reverse: 5’- GCCGTGGTGACGCCGTACT-3’.

### RNA preparation and real-time PCR

RNA quantification, cDNA preparation, and real-time PCR were performed as discussed previously [18]. Briefly, total RNA was isolated from cells by Tri reagent (Ambion, Thermo Fischer Scientific, USA). The concentration and purity of RNA were determined by Nanodrop (Thermo Fischer, USA). Two micrograms of RNA were treated with RNase-free DNase (Promega, USA), and subsequently reversed transcribed into cDNA by using the First-strand cDNA synthesis kit (Thermo Scientific, USA), as per the manufacturers’ instructions. Real-time PCR was performed on the resulting cDNA with gene-specific primers (*LdAAP3*: Forward 5’ CGGTCGAAATGGTGCCAAAC 3’, Reverse 5’ GGCTTCATCTTCCCTGCGTA 3’; *LdPT*: Forward 5’ AGGACGCTGCTCAACTCTTC 3’, Reverse 5’ AAGGCGAACGTGTCACTCAA 3’; kinetoplast minicircle DNA (control) [19]: JW11 5’ CCTATTTTACACCAACCCCCAGT 3’; JW12 5’ GGGTAGGGGCGTTCTGCGAAA 3’) using the PowerUp SYBR Green Master Mix (Applied Biosystems, USA). The PCR amplification program used was as follows: 50°C for 2 minutes (min) and 95°C for 10 seconds (sec), followed by 40 cycles at 95°C for 15 sec, 59°C for 1 min, and 72°C for 20 sec. The amplification of the kinetoplast minicircle DNA of *L*. *donovani* was used as an internal control. The results were expressed as fold change of control (2 h infected cells) using the method described by Pfaffl, 2001 [20]. The real time PCR primers did not amplify any products in uninfected macrophages. All sample analysis was performed in triplicate, and each experiment was performed three times.

### Cell viability assay

At the specified times following infection or treatment with inhibitors, THP-1 cells were washed two times with PBS. MTT [3-(4,5-dimethyl-2-thizolyl)-2,5-diphenyltetrazolium bromide] dye solution (Sigma-Aldrich, USA) (5 mg MTT in 1 ml PBS) was diluted 1:10 in RPMI medium (normal or with 0.1 mM arginine). Uninfected or infected THP-1 cells were incubated in diluted MTT dye solution at 37°C in a 5% CO_2_-air atmosphere for 2 h and thereafter incubated with stopping solution which consisted of isopropanol containing 5% formic acid, at 150 rpm, 37°C for 20 min. *L*. *donovani* promastigotes were incubated with diluted MTT dye solution for 3 h at 37°C, and incubated with stopping solution comprising of isopropanol and 20% SDS in a 1:1 ratio, at 80 rpm, 37°C for 30 min. Absorption was then measured at 570 nm, and the percentage cell viability was calculated.

### Infectivity assay

THP-1 cells were seeded on glass coverslips (1 x 10^6^ cells/well) in a 6-well plate and treated with 50 ng/ml of PMA (Sigma-Aldrich, USA) for 48 h. They were infected as described above, and the intracellular parasite load was visualized by Giemsa staining.

### Western blotting

Western blot analysis of LdAAP3 was performed as described previously by Darlyuk et al., 2009 [17].

### Statistical analysis

Real-time PCR data were analyzed by GraphPad prism and represented as mean ± standard error of the mean (S.E.M.). Student’s unpaired 2-tailed t-test was used to calculate significance. *P* value < 0.01–0.05 was considered statistically significant (*), *p* < 0.001–0.01 was considered very significant (**), and *p* < 0.0001–0.001 was considered extremely significant (***). The northern blot image data was analyzed using ImageJ analysis software. Data from three independent experiments were analyzed and represented as mean ± standard error of the mean (S.E.M.).

## Results

### Low arginine concentration activates arginine deprivation response (ADR) in intracellular *Leishmania donovani*

**Fig 1:**
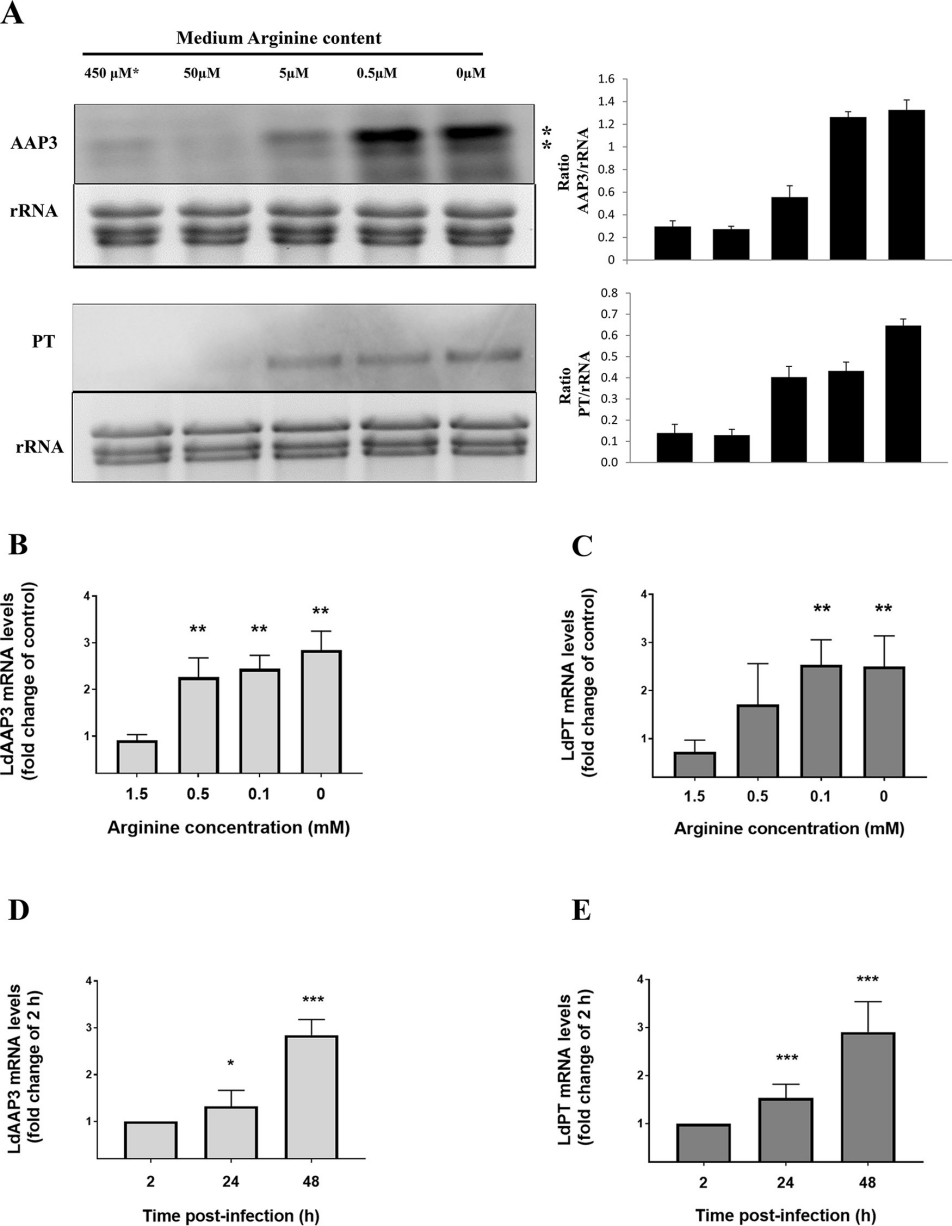
The activation of ADR is arginine concentration-dependent. A. The minimal arginine concentration required for activation of ADR in axenic promastigotes. The promastigotes were cultured either in M199 medium containing 0.45 mM arginine or in arginine deprived M199 medium containing different concentrations of arginine (0.5 μM, 5 μM and 50 μM). The promastigotes were cultured in the respective medium for two hours, the total RNA was isolated and Northern blot analysis was carried out. Changes in the LdAAP3 and LdPT expression levels were determined by gene specific probes as described in Materials and Methods. Data are representative of three independent experiments. The two asterisks (**) in the figures refer to the two transcripts (5-and 7-kb) synthesized from the LdBPK_310910.1 gene. B and C. The minimum concentration of arginine required for ADR activation in intracellular amastigotes. THP-1 cells were infected with L. donovani in RPMI medium containing 0 (actual arginine concentration in RPMI medium is 0.008 mM), 0.1 mM, 0.5 mM or 1.5 mM arginine for 48 h. The total RNA was then extracted, and the resulting cDNA was subjected to real-time PCR analysis using primers specific for LdAAP3 (B) and LdPT (C). The results are expressed as fold-change of control (2 h infected cells). Values are mean ± S.E.M. The results are representative of three independent experiments performed in triplicates. **, p < 0.001–0.01. The real time PCR primers do not amplify any products (LdAAP3 or LdPT) in uninfected macrophages. D and E. Time-course of ADR. THP-1 cells were infected with L. donovani in RPMI medium containing 0.1 mM arginine for 2 h, 24 h and 48 h. The total RNA was then extracted, and the resulting cDNA was subjected to real-time PCR analysis using primers specific for LdAAP3 (D) and LdPT (E). The results are expressed as fold-change of control (2 h infected cells). Values are mean ± S.E.M. The results are representative of three independent experiments performed in triplicates. *, p < 0.01–0.05, **, p < 0.001–0.01, ***, p < 0.0001–0.001. The real time PCR primers did not amplify any products (LdAAP3 or LdPT) in uninfected macrophages.

The first set of experiments aimed to identify the basal concentration of arginine required for the activation of the ADR in *L*. *donovani* promastigotes. The maximal concentration of arginine required for ADR activation was found to be 5 μM, which resulted in the up-regulation of Ld*AAP3* and threshold was lower at 0.5 μM for the pteridine transporter (Ld*PT*) at mRNA level (Fig 1A). However, unlike *LdAAP3* the expression of *LdPT* mRNA was not linear dose dependent. Interestingly, this concentration of arginine is close to the apparent Km value of 2.4 μM for *L*. *donovani* LdAAP3 transport activity [8], raising the question whether this transporter is also the sensor.

Previously, we observed that 48 hours after infecting THP-1 macrophages, the expression of Ld*AAP3* in intracellular *L*. *donovani* increased almost two-fold as compared to promastigotes [7]. This suggested that during development inside phagolysosomes, parasites encountered a low level of arginine that activated ADR. In this case, increasing external arginine concentrations might help to maintain the phagolysosome arginine concentration above the threshold. To test this, we infected THP-1 macrophages that grew in media containing 0, 0.1, 0.5 and 1.5 mM arginine. At 48 hours post-infection, total RNA was extracted from infected macrophages, and the resulting cDNA was subjected to real-time PCR, using Ld*AAP3* and Ld*PT* primers as probes, as mentioned in the methods section (Fig 1B and 1C, respectively). As shown, the mRNA abundance of both genes increased as arginine concentration in the medium decreased. The results indicate that the arginine concentration in phagolysosomes of macrophages grown in a medium that contains arginine at a concentration of 0.1 mM and below activates ADR. A literature search indicated that the arginine concentration in human blood is ~80 μM [21], thereby indicating that ADR activation in our experiments was achieved under physiological conditions. Additionally, the infectivity of *L*. *donovani* in THP-1 cells cultured in media containing different concentrations of arginine was ~40% (S2A Fig).

As control, THP-1 cells, either uninfected or infected with *L*. *donovani* in medium containing different concentrations of arginine were subjected to an MTT assay to determine their viability at 48 hours. As seen in S1A Fig, the macrophages were 85–100% viable in media containing different arginine concentrations, thus indicating that ADR activation in intracellular amastigotes was not detrimental to macrophage viability. Additionally, the expression of *LdAAP3* remained unchanged in *L*. *donovani* promastigotes cultured in media containing 0.1 mM and 0.5 mM arginine (S1B Fig), and promastigote viability was between 90–100% (S1C Fig). This proves that extracellular parasites did not contribute to the observed activation of ADR in intracellular amastigotes.

**Fig 2:**
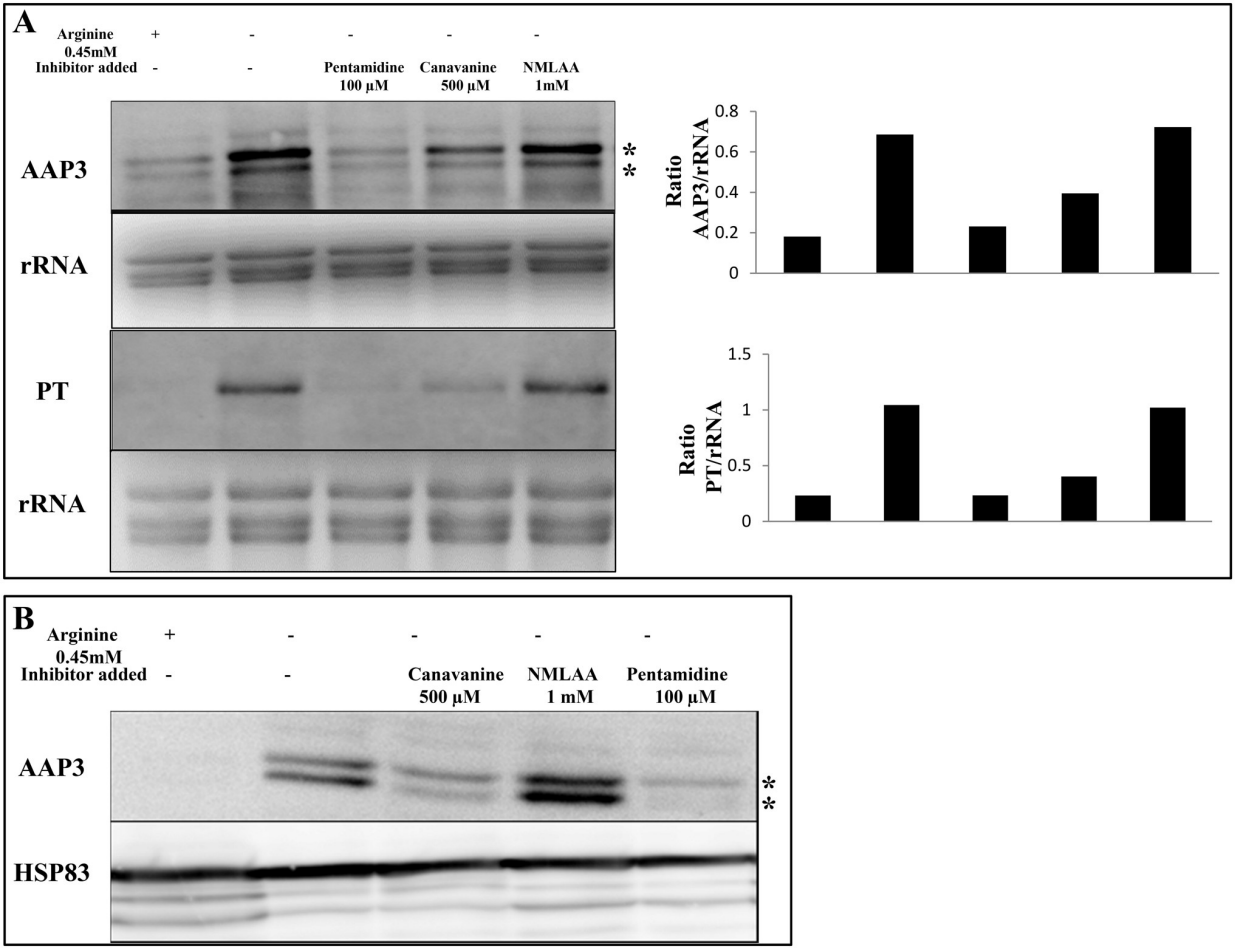
Effect of arginine transport inhibitors on ADR. A. The effect of three different arginine transport inhibitors was checked on ADR. Axenic promastigotes were cultured in M199 medium and the cells were washed with Earl’s salt solution followed by starvation for two hours in arginine deprived M199 medium in the presence of 100 μM pentamidine, 500 μM canavanine and 1 mM NMLAA. Post-starvation, the total RNA was isolated and Northern blot analysis was carried out. The effect of transport inhibitors on ADR was checked by determining the expression levels of LdAAP3 and LdPT using gene specific probes as described in Materials and Methods. Line about histogram should be inserted here. The two asterisks (**) in the figures refer to the two transcripts (5-and 7-kb) synthesized from the LdBPK_310910.1 gene. B. Western blot analysis of the effect of arginine transport inhibitors on ADR. Data are representative of three independent experiments.

ADR activation in intracellular amastigotes was also determined in a time-course analysis of the infection cycle. THP-1 cells were infected with *L*. *donovani* in medium containing 0.1 mM arginine for 2 h, 24 h and 48 h post-infection and harvested at the end of each time point for RNA analysis (Fig 1D and 1E). Real-time PCR showed that the up-regulation of Ld*AAP3* and Ld*PT* started at 24 h post-infection and continued to increase at 48 h post-infection, thereby implying that the activation of ADR in intracellular amastigotes occurs between 24–48 hours post-infection. This infers that the initial arginine concentration in infected phagolysosomes is high and reduces with time, reaching ADR activation level at 24 h post-infection. However, it could also be possible that induction of the ADR is delayed due to the time taken to deplete intracellular parasite pools of arginine following their phagocytosis. As seen in Fig 2B, the infectivity of *L*. *donovani* in THP-1 cells at 2 h, 24 h and 48 h post-infection was between 28–38%.

Previous studies have indicated that arginine concentration in the mammalian lysosomes is higher than that in their cytosol [22] and in *Saccharomyces cerevisiae* vacuoles [23]. Because lysosome volume was not determined in this study it did not provide accurate arginine concentrations. We have now determined the arginine concentration in THP-1 macrophage phagolysosomes that seems to agree with the observation of Harms *et al*. (Fischer-Weinberger *et al*, in preparation). The initial arginine concentration in THP-1 phagolysosomes is 0.14 mM, a concentration that is higher than the concentration we found that activates ADR in axenic parasites. This further supports our findings that the late activation of ADR during infection is due to the time it takes for intra-phagolysosome parasites to utilize arginine and reduce its concentration to ≤5 μM.

### Effect of arginine analogues on ADR

Arginine is a positively charged amino acid with an amidino group at the distal cap of its side chain. We hypothesized that this side chain of arginine is the ligand that binds the arginine sensor and transporter on the parasite surface. To determine this, we first analyzed various structural L-arginine analogues which were previously shown to be arginine transport inhibitors [24,25]. Canavanine has a guanidinoxy group with a conserved amidino group as in arginine, and N-methyl L-arginine acetate (NMLAA) is another structural analogue of L-arginine where the amidino group is modified by the addition of a methyl group. The methyl-amidino group of NMLAA has a net charge of zero at pH 7. NMLAA has been previously shown to be an arginine transport inhibitor in *L*. *donovani* [24] and canavanine inhibits arginine transport in *S*. *cerevisiae* [26,27] and *T*. *brucei* [25]. To test the effect of these structural analogues on ADR, we determined the minimal concentration of each structural analogue that upon two hours treatment inhibited arginine transport in promastigotes but had no effect on cell viability (Table 1). This list includes 0.5 mM canavanine, and 1 mM NMLAA (left column). We determined the effect of these compounds on ADR activation in promastigotes. This was carried out in arginine-depleted *L*. *donovani* axenic promastigotes cultured in M199 with or without arginine transport inhibitors followed by Northern blot analysis using gene-specific probes for *LdAAP3* and *LdPT*. Fig 2A shows that Canavanine inhibited *LdAAP3* (27%±1.9) and *LdPT* (61%±2.4) mRNA up-regulation. In contrast, NMLAA did not have any inhibitory effect on ADR as the expression of both the genes was found to be up-regulated upon arginine deprivation (Fig 2A). Identical results were obtained at the protein level for LdAAP3 in a Western blot analysis for cananvanine and NMLAA (Fig 2B). Hence, methylation of the amidino group in NMLAA retained binding to the LdAAP3 transporter but lost recognition by the sensor.

**Table 1:** Arginine transport inhibition by arginine and side chain analogues.

Compound	Concentration*	Percent arginine transport inhibition
1) L-Arginine	25 μM	100%
2) Pentamidine	100 μM	38%
3) N-Methyl L-Arginine Acetate	1 mM	66%
4) Canavanine	500 μM	40%
5) Nω-Nitro-L-arginine methyl ester	1 mM	25% ^#^
6) Nω-Nitro-L-arginine	1 mM	23% ^#^
7) 4-{[5-(4-aminophenoxy)pentyl]oxy}Phenylamine	100 μM	n.d.
8) Ureidopropionic acid	1 mM	n.d.
9) L-citrulline	1 mM	10%
10) D-Arginine	1 mM	20% ^#^

*Maximal concentration that inhibited arginine transport. Indicated inhibitor concentrations were the maximal non-toxic values and did not affect parasite viability. Arginine transport rate: 26.13 ± 2.97 pmol of L-arginine per minute per 10^6^ cells. Transport inhibitors were added at a concentration of 100 μM (four fold excess over 25 μM arginine) or 500 μM (20-fold excess) or 1 mM (40-fold excess).

^#^ indicates that these compounds were previously studied as arginine transport inhibitors in *L*. *donovani* and their percent arginine transport inhibition were determined from a published study [24].

**Fig 3:**
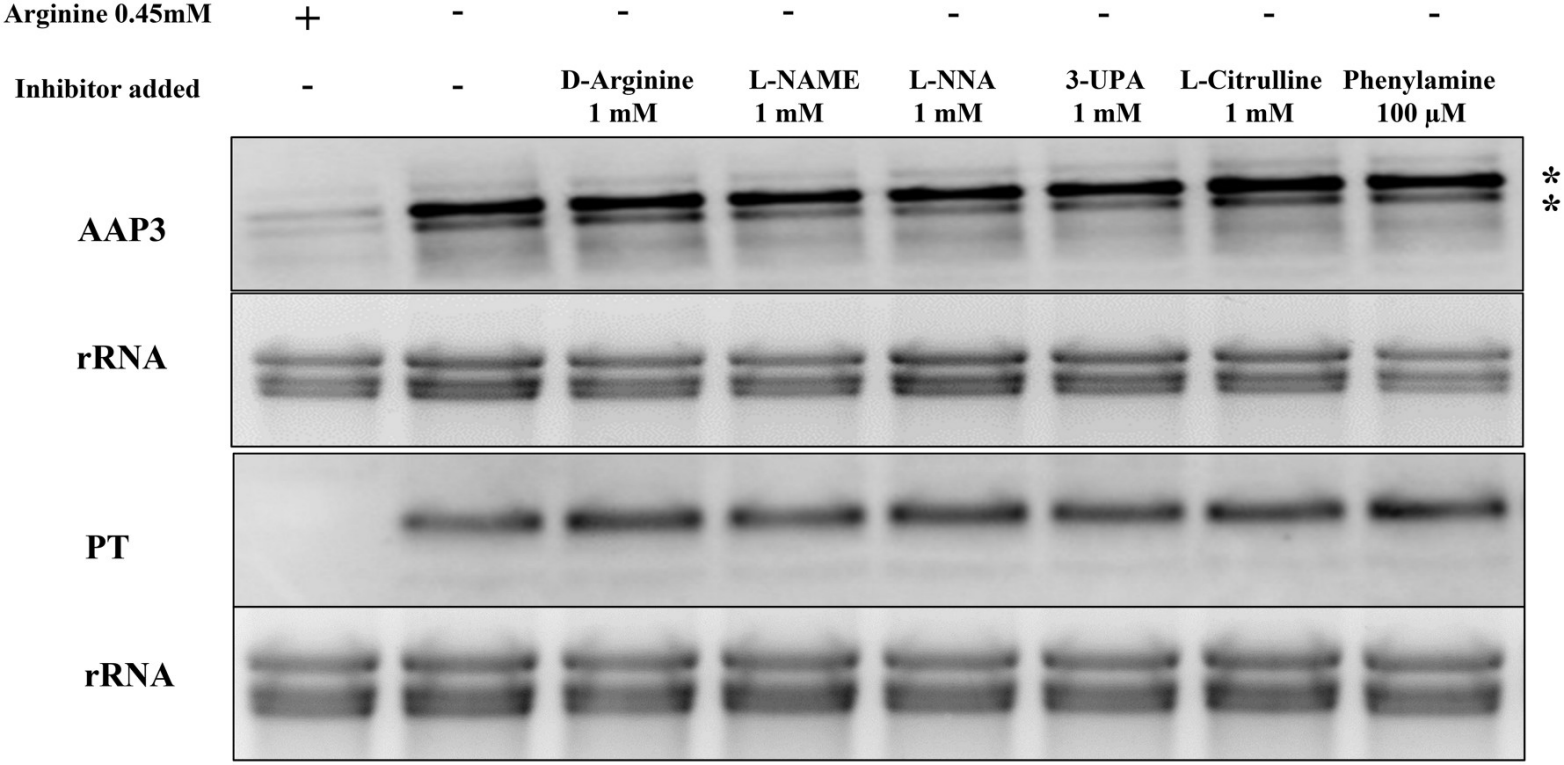
Effect of amidino-group modified arginine analogues and compounds on ADR. A. The effect of six different arginine analogues or compounds was checked on ADR. Axenic promastigotes were cultured in M199 medium and the cells were washed with Earl’s salt solution followed by starvation for two hours in arginine deprived M199 medium in the presence of 1 mM D-Arginine, 1 mM Nω-Nitro-L-arginine methyl ester (L-NAME), 1 mM Nω-Nitro-L-arginine (L-NNA), 1 mM 3-Ureidopropionic acid (UPA), 1 mM L-citrulline and 100 μM 4-{[5-(4-aminophenoxy)pentyl]oxy}phenylamine. Post-starvation, the total RNA was isolated and Northern blot analysis was carried out. The effect of transport inhibitors on ADR was checked by determining the expression levels of LdAAP3 and LdPT using gene specific probes as described in Materials and Methods. Data are representative of three independent experiments. The two asterisks (**) in the figures refer to the two transcripts (5-and 7-kb) synthesized from the LdBPK_310910.1 gene.

Further, the effect of the enantiomer D-arginine as compared to L-arginine on ADR was determined. D-arginine is not a competitive inhibitor of L-arginine transport [24]. This was carried out in *L*. *donovani* promastigotes in M199 medium with or without D-arginine (1 mM) and Northern blot was performed as mentioned above. Fig 3 shows that D-arginine had no effect on ADR and did not lead to the degradation of *LdAAP3* and *LdPT* mRNA. This indicated that, the external arginine sensor in the parasite could distinguish between D-arginine and L-arginine.

We further checked what is the effect of chemical compounds that have a conserved amidinio side chain group of arginine and one such compound was pentamidine. Pentamidine is a diamidine that has two amidino moieties previously shown to be competitive arginine transport inhibitors in *L*. *donovani* [8,28]. These amindino groups have a net positive at pH7. Analysis of the effect of 100 μM pentamidine on axenic promastigotes showed that pentamidine drastically inhibited both *LdAAP3* (81%±2.8) and *LdPT* (72%±3.2) up-regulation at 2 h post arginine deprivation (Fig 2A). This suggests that the minimal molecular group necessary for recognition by the surface arginine sensor and transporter binding sites is the amindino group of the arginine side chain. Similar result was also seen in Western blot analysis where pentamidine inhibited the ADR-stimulated expression of LdAAP3 (Fig 2B). This minimal amidino group of the arginine side chain is sufficient to inhibit ADR and arginine transport and it is direct proof that the α-carbon group is not necessary for arginine sensing as well as transport.

**Fig 4:**
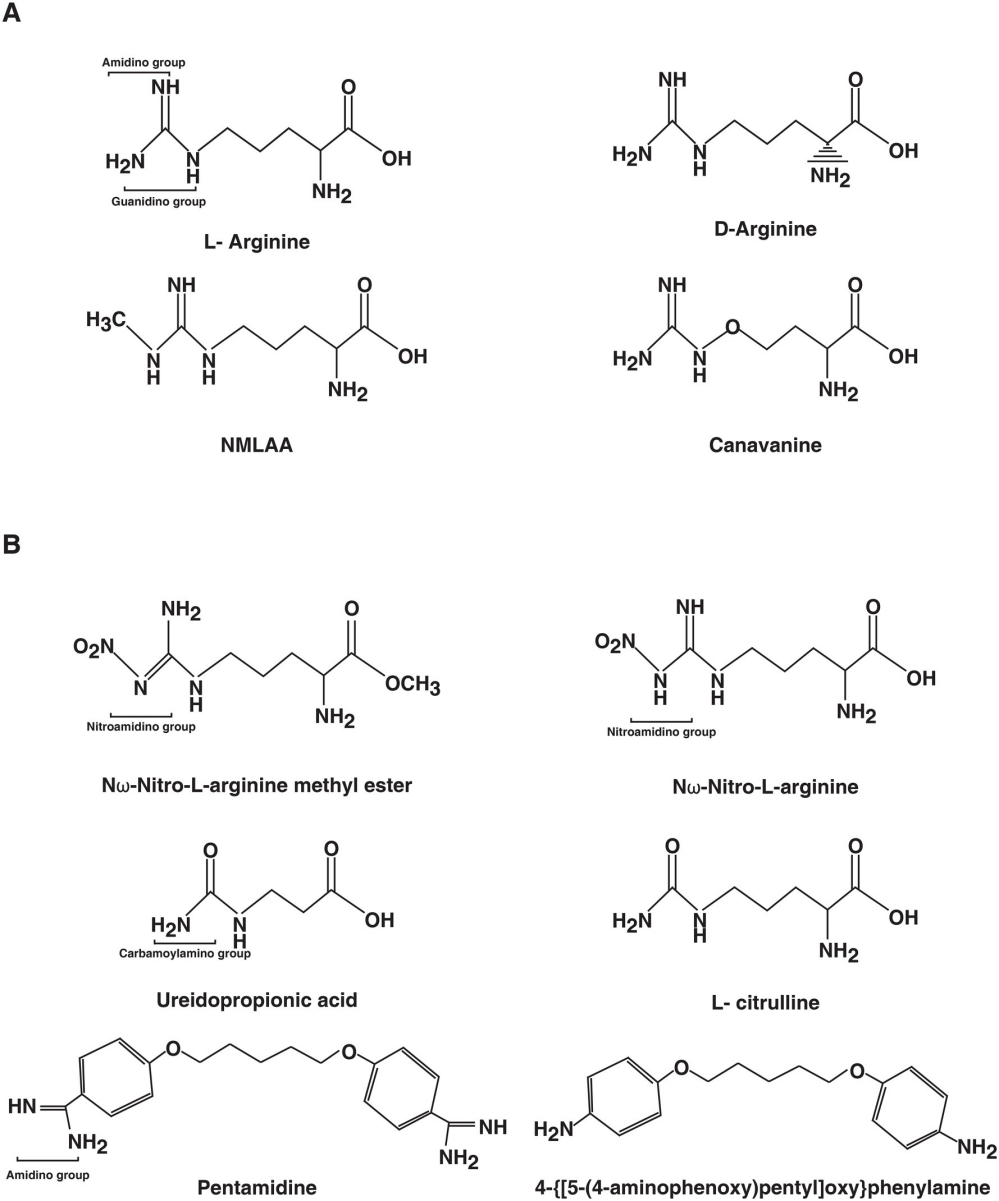
Molecular structures of arginine analogues. (A) Molecular structure of arginine structural analogues (D-Arginine, NMLAA and Canavanine) used in the present study. (B) Molecular structure of arginine side chain analogues.

Further, the analogues of L-arginine which have a modified amidino/guanidine group (like NMLAA) and their effect on ADR was analyzed. Nω-Nitro-L-arginine methyl ester (L-NAME) and Nω-Nitro-L-arginine (L-NNA) are arginine analogues where the amidino group is modified by the addition of a nitro-group (nitro-amidino). The net charge of the nitroguanidinium group in L-NAME is neutral and the primary α-amino group is positively charged [29], while the gaunidino group of L-arginine has a net positive charge. Both arginine analogues have been previously shown to be weak arginine transport inhibitors in *L*. *donovani* [24]. In addition to the above mentioned arginine transport inhibitors, other compounds which have a deamidated guanidino group were also tested for their effect on ADR. L-citrulline is an alpha amino acid and an intermediate in the urea cycle. It has a carbamoylamino group which is formed by the deamination of the guanidium group, and has previously been shown by our group to be a poor arginine transport inhibitor for LdAAP3 [8]. Citrulline is known to have no net charge at pH 7 unlike arginine which has a net positive charge. Similarly, 3-Ureidopropionic acid has an carbamoylamino group similar to citrulline but has a missing primary amine. Additionally, a pentamidine analogue 4-{[5-(4-aminophenoxy)pentyl]oxy}phenylamine, which has the two amidino groups of pentamidine substituted with amines was also analyzed. All these compounds were used further to test their effect on ADR in *L*. *donovani* (see all the molecular structures in Fig 4).

There was no inhibition of *LdAAP3* and *LdPT* levels when *L*. *donovani* promastigotes were treated with the above mentioned compounds (Fig 3). This indicates that there are two distinct arginine binding sites in the ADR machinery in *L*. *donovani*. The complete list of molecular structures of all the arginine analogues (structural and side chain analogues) is shown in Fig 4. These compounds were not tested for the regulation of LdAAP3 and LdPT at the protein level in promastigotes and at the RNA and protein levels in intracellular amastigotes as their effect at the RNA level was similar to that of NMLAA. Thus, NMLAA and other compounds with a modified guanidino/amindino group have a net charge of zero as compared to the net positive charge of the guanidino/amindino group of arginine. This suggests that in addition to the presence of a functional amidino or guanidino group, the positive charge on the R-group of arginine is also an important factor for the recognition of arginine by the *L*. *donovani* sensor.

### Effect of exogenous arginine on arginine sufficiency in *L*. *donovani* promastigotes and intracellular amastigotes

**Fig 5:**
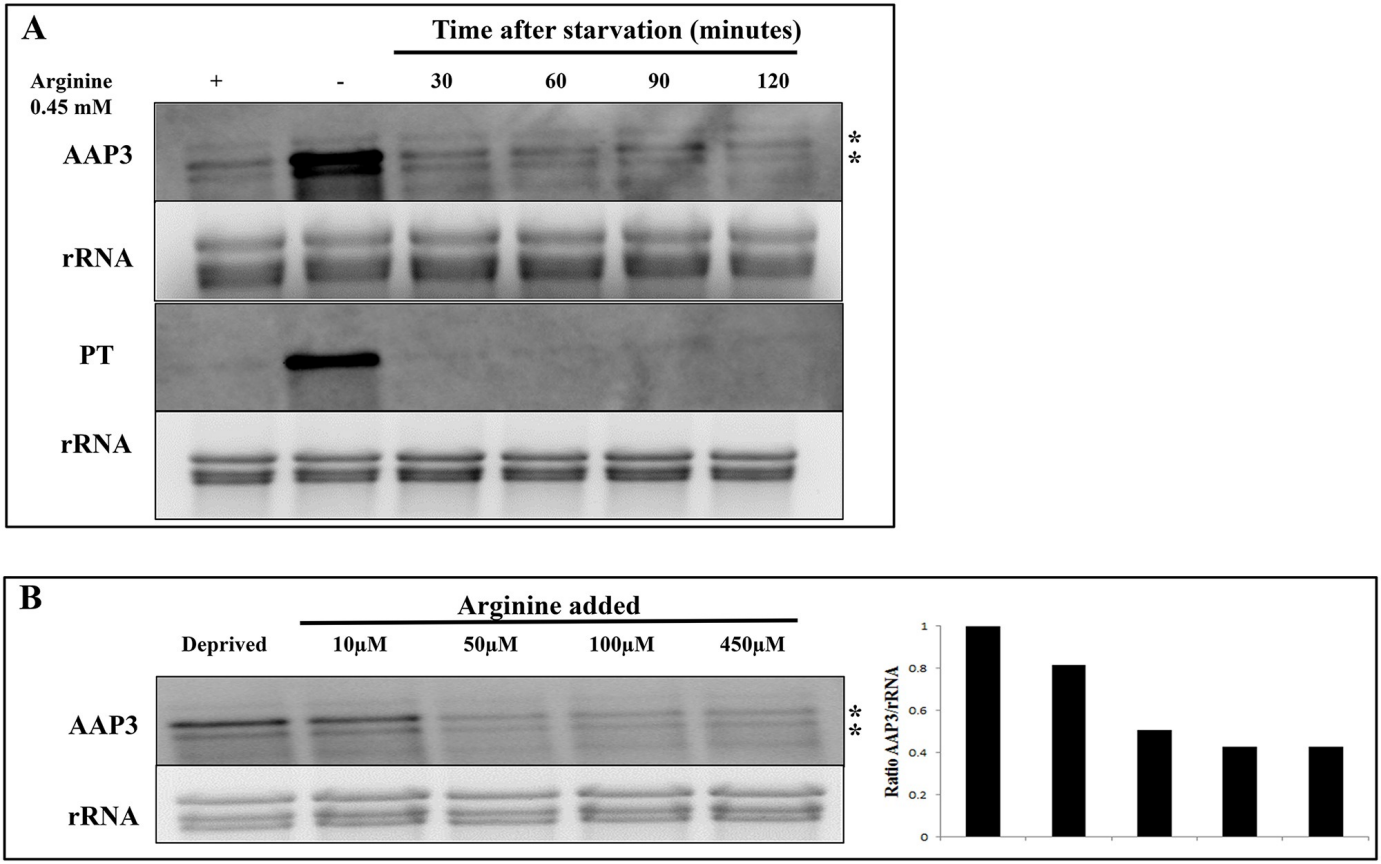
Time course analysis of the effect of exogenous arginine on ADR in promastigotes. The effect of addition of exogenous arginine on ADR was checked. Axenic promastigotes were cultured in M199 medium and the cells were washed with Earl’s salt solution followed by starvation for two hours in arginine deprived M199 medium. After two hours, exogenous arginine (0.45 mM) was added to the arginine deprived cells and the cells were further incubated for the following time periods (30 min, 60 min, 90 min and 120 min) after starvation. The total RNA was isolated from undeprived and arginine deprived cells. The effect of exogenous arginine on ADR was checked by determining the expression levels of LdAAP3 and LdPT using gene specific probe as described in Materials and Methods. B. The minimal concentration required for activating arginine sufficiency signaling via arginine sensor was determined. Axenic promastigotes were cultured in M199 medium and the cells were washed with Earl’s salt solution followed by starvation for two hours in arginine deprived M199 medium. After two hours, exogenous arginine (10 μM, 50 μM, 100 μM and 450 μM) was added to the arginine deprived cells, and the cells were further incubated for one hour. The total RNA was isolated from undeprived and arginine deprived cells. The effect of exogenous arginine on ADR was checked by determining the expression levels of LdAAP3 transporter using a gene-specific probe. The abundance of expression of LdAAP3 was normalized relative to the rRNA of each condition, and this was calculated using ImageJ program and is illustrated in the graph below the gel. Data are representative of three independent experiments. The two asterisks (**) in the figures refer to the two transcripts (5-and 7-kb) synthesized from the LdBPK_310910.1 gene.

We have previously reported that the arginine sensor responds to both arginine deprivation and sufficiency and the addition of exogenous arginine to two hours arginine starved axenic promastigotes induces rapid degradation of the LdAAP3 protein to the level observed in un-deprived cells [7]. In order to further analyze arginine sufficiency response at the mRNA level, exogenous arginine (0.45 mM) was added to two hours arginine deprived axenic promastigotes which induced the rapid degradation of Ld*AAP3* (rate of t_1/2_ = 30 minutes) and Ld*PT* mRNA (Fig 5A). This implies that the regulation of the LdAAP3 degradation signal occurs not only at the protein level as previously suggested [7], but also at the post-transcriptional level. The minimal threshold of arginine sufficient to be detected by the arginine sensor as arginine sufficiency and thereby downregulate ADR was also checked. However, *LdPT* mRNA was degraded more rapidly as seen in Fig 5A and did not follow the same mRNA degradation kinetics as *LdAAP3*. It was observed that 10 μM exogenous arginine did not activate arginine sufficiency signaling via arginine sensor, but 50 μM, 100 μM and 450 μM of exogenous arginine were detected as arginine sufficiency, thereby resulting in the downregulation of Ld*AAP3* levels (Fig 5B).

**Fig 6:**
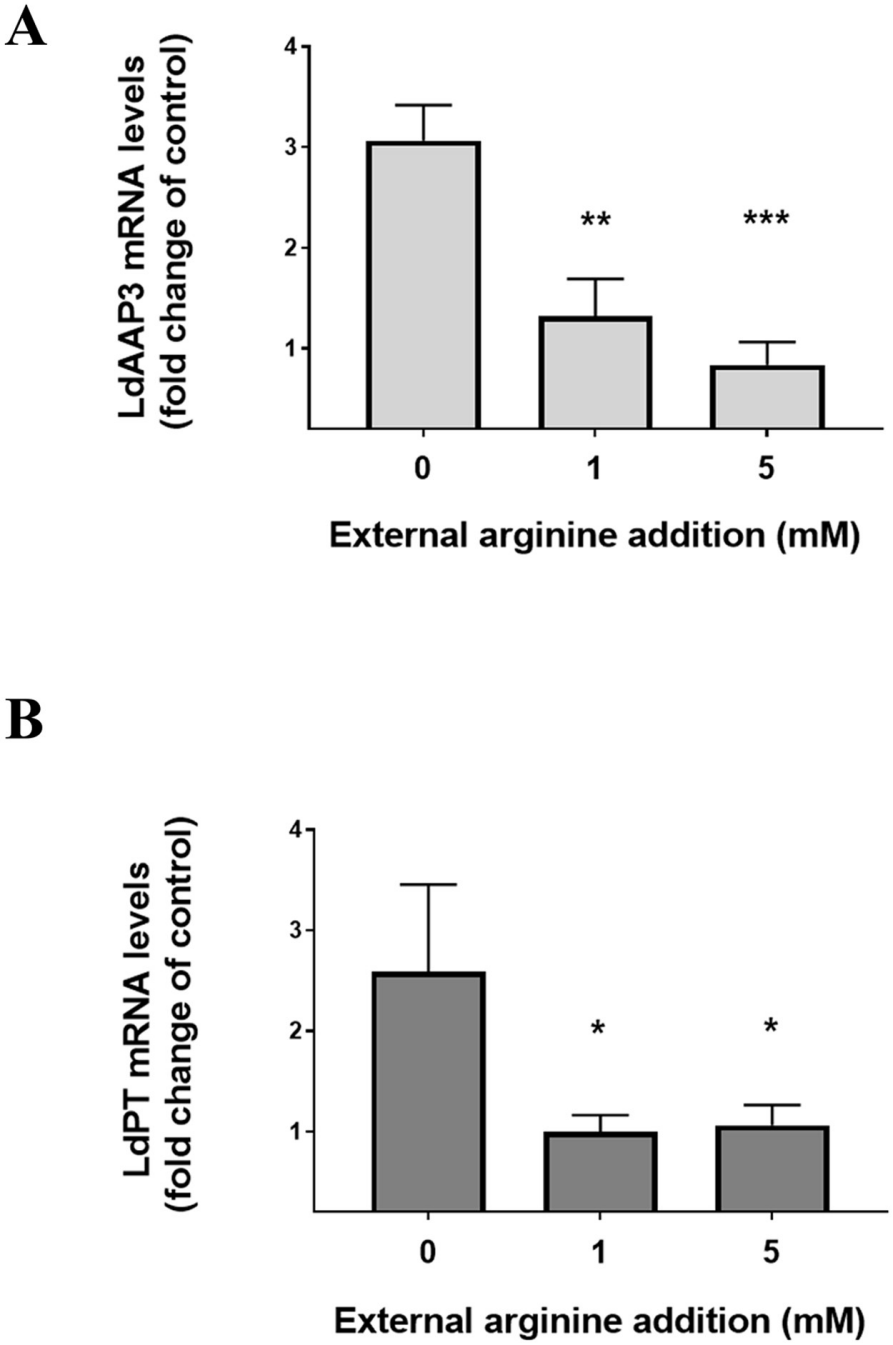
The addition of external arginine to intracellular amastigotes induces rapid degradation of LdAAP3 and LdPT. A and B. LdAAP3 and LdPT levels of intracellular amastigotes treated with external arginine. THP-1 cells were infected with L. donovani in RPMI medium containing 0.1 mM arginine for 48 h. They were then treated with 1 mM or 5 mM of arginine for 2 h. The total RNA was extracted, and the resulting cDNA was subjected to real-time PCR analysis using primers specific for LdAAP3 (A) and LdPT (B). The results are expressed as fold-change of control (2 h infected and untreated cells). Values are mean ± S.E.M. (n = 3). The results are representative of three independent experiments performed in triplicates.

In order to ascertain whether the arginine sufficiency phenomenon also occurs in intracellular amastigotes, THP-1 macrophages were infected with *L*. *donovani* for 48 h in medium containing 0.1 mM arginine, and then excess arginine (1 mM or 5 mM) was added for an additional 2 h. RNA was extracted from infected macrophages before and after the addition of excess arginine and subjected to real-time PCR. This resulted in the down-regulation of Ld*AAP3* and Ld*PT* mRNA in intracellular amastigotes (Fig 6A and 6B). This indicated that intracellular amastigotes, like the axenic, respond to arginine sufficiency by rapidly down-regulating ADR. As seen in ***Supp***. Fig 2C, the differences in infectivity of *L*. *donovani* in THP-1 cells treated ot not with exogenous arginine were not significant.

### Effect of arginine analogues on arginine sufficiency in *L*. *donovani*

**Fig 7:**
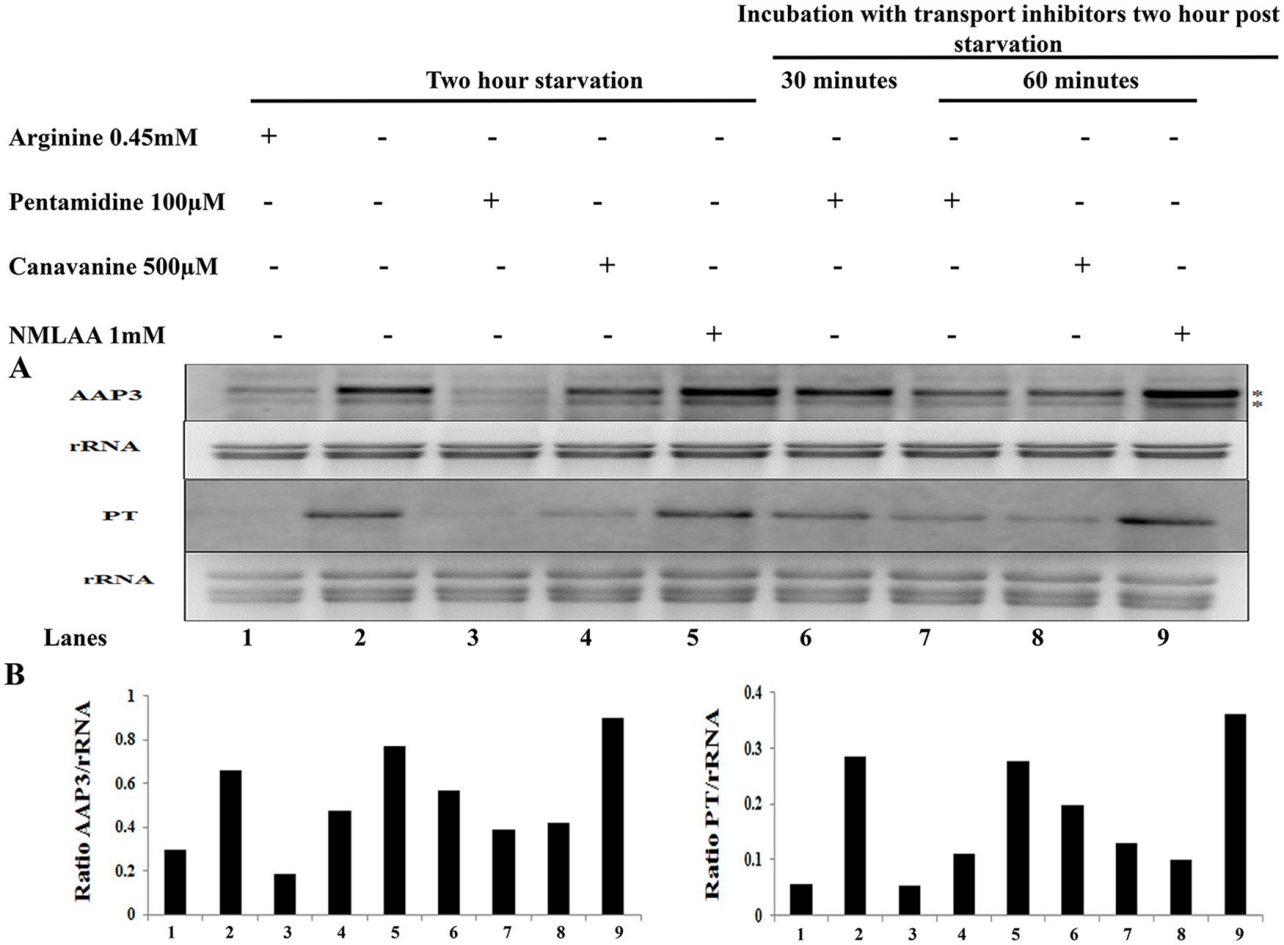
Arginine transport inhibitors pentamidine and canavanine down-regulate ADR by the rapid degradation of LdAAP3 and LdPT. A and B. The effect of arginine transport inhibitors on ADR was checked. Axenic promastigotes were cultured in an M199 medium, and the cells were washed with Earl’s salt solution followed by starvation for two hours in arginine deprived M199 medium. After two hours, arginine transport inhibitors (100 μM pentamidine, 500 μM canavanine and 1 mM NMLAA) were added to the arginine deprived cells, and the cells were further incubated for the following time periods (30 min and 60 min for pentamidine; 60 min respectively for canavanine and NMLAA). The total RNA was isolated from undeprived and arginine deprived cells. The effect of transport inhibitors on ADR was checked by determining the expression levels of LdAAP3 (A) and LdPT (B) using gene-specific probes as described in Materials and Methods. The abundance of the expression of LdAAP3 or LdPT normalized relative to the rRNA of each condition was calculated using ImageJ and is illustrated in the graphs below the gels. The image is representative of three independent experiments. The two asterisks (**) in the figures refer to the two transcripts (5-and 7-kb) synthesized from the LdBPK_310910.1 gene.

**Fig 8:**
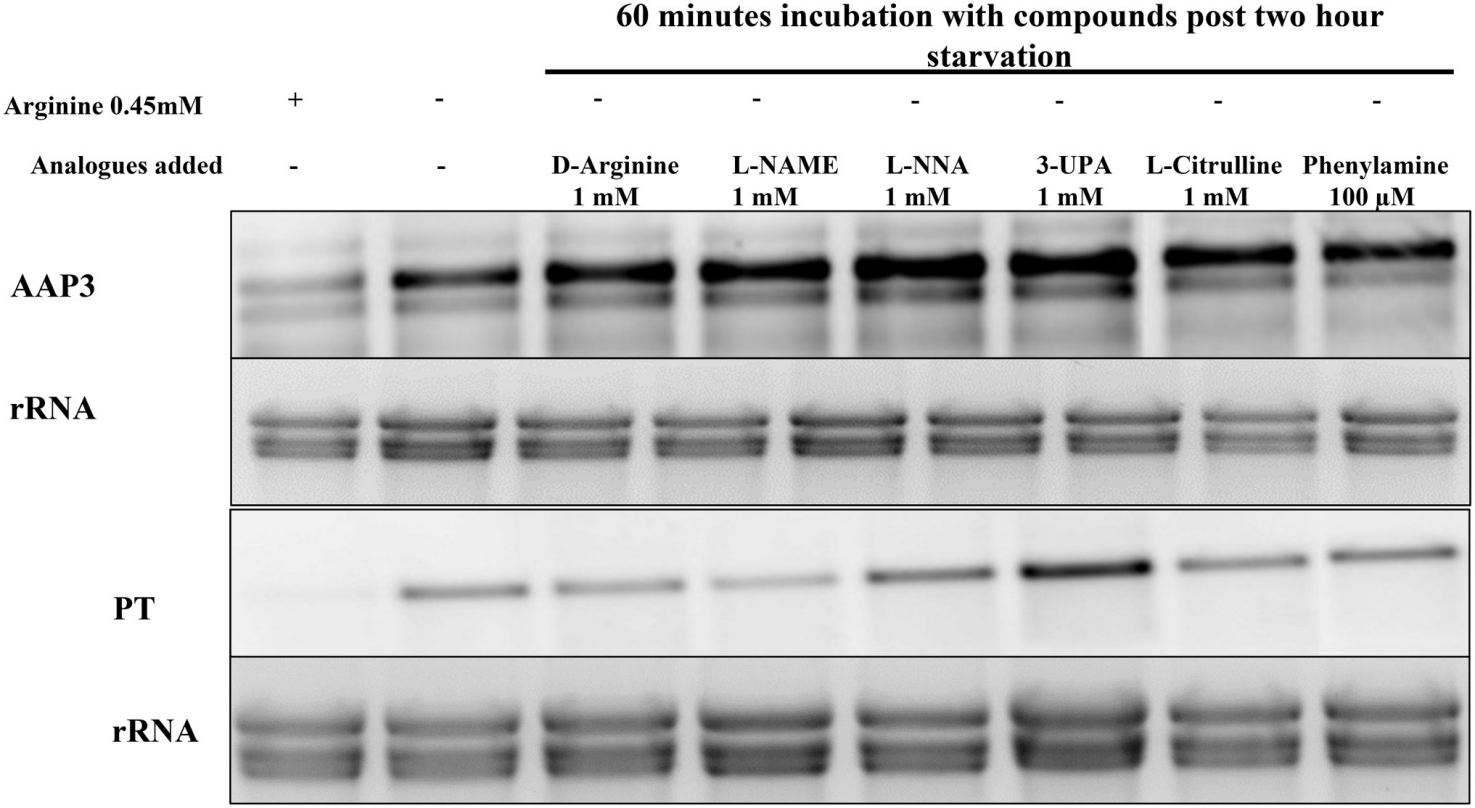
Amidino-group modified arginine analogues and compounds don’t affect arginine sufficiency and ADR by the rapid degradation of LdAAP3 and LdPT. A. The effect of amidino-group modified arginine analogues and compounds on ADR was checked. Axenic promastigotes were cultured in M199 medium, and the cells were washed with Earl’s salt solution followed by starvation for two hours in arginine deprived M199 medium. After two hours, amidino-group modified arginine analogues and compounds (1 mM D-Arginine, 1 mM Nω-Nitro-L-arginine methyl ester (L-NAME), 1 mM Nω-Nitro-L-arginine (L-NNA), 1 mM 3-Ureidopropionic acid (UPA), 1 mM L-citrulline and 100 μM 4-{[5-(4-aminophenoxy)pentyl]oxy}phenylamine) were added to the arginine deprived cells, and the cells were further incubated for 60 minutes. The total RNA was isolated, and the effect of amidino-group modified arginine analogues and compounds on ADR was checked by determining the expression levels of LdAAP3 and LdPT using gene-specific probes as described in Materials and Methods. The abundance of the expression of LdAAP3 or LdPT normalized relative to the rRNA of each condition was calculated using ImageJ and is illustrated in the graphs below the gels. The image is representative of three independent experiments. The two asterisks (**) in the figures refer to the two transcripts (5-and 7-kb) synthesized from the LdBPK_310910.1 gene.

As it was observed that the arginine transport inhibitors (pentamidine and canavanine) inhibited ADR, we subsequently checked the effect of these inhibitors on arginine sufficiency. *L*. *donovani* axenic promastigotes were incubated for 2 h in M199 medium lacking arginine. Thereafter, arginine transport inhibitors (pentamidine (100μM), canavanine (500μM), NMLAA (1mM), D-arginine (1mM), L-NAME (1 mM), L-NNA(1 mM), L-citrulline (1 mM), 3-Ureidopropionic acid (1 mM) and 4-{[5-(4-aminophenoxy)pentyl]oxy}-phenylamine (100 μM)) were added to arginine deprived cells, and the cells were harvested at different time-points. Upon Northern blot analysis, it was observed that only pentamidine and canavanine down-regulated ADR as evidenced by the rapid degradation of LdAAP3 (Fig 7A) and LdPT (Fig 7B) in a time-dependent manner. However, NMLAA(Fig 7A) and the other analogues (Fig 8) had no effect on ADR.

### Effect of arginine analogues on arginine sufficiency in intracellular *L*. *donovani*

**Fig 9:**
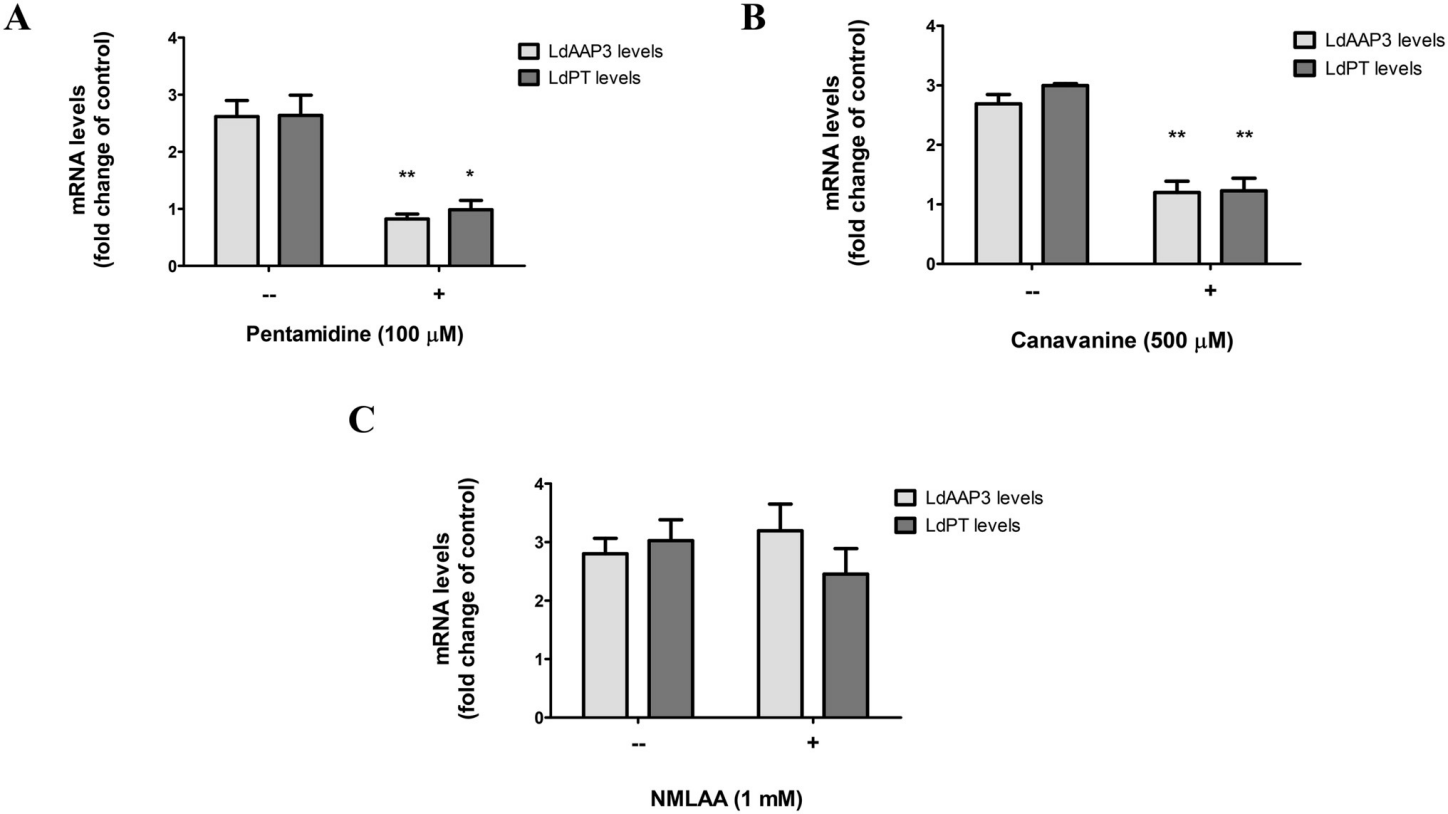
Pentamidine and canavanine inhibit ADR in intracellular amastigotes, while NMLAA does not. A–C. Real-time PCR to monitor LdAAP3 and LdPT levels in intracellular amastigotes treated with arginine analogues. THP-1 cells infected with L. donovani in RPMI medium containing 0.1 mM arginine for 48 h were treated with 100 μM pentamidine (A), 500 μM canavanine (B), or 1 mM NMLAA (C) for 2 h. Infected and untreated cells were used as control. The total RNA was extracted, and the resulting cDNA was subjected to real-time PCR analysis using primers specific for LdAAP3 and LdPT. The results are expressed as fold-change of control (2 h infected and untreated cells). Values are mean ± S.E.M. (n = 3). The results are representative of three independent experiments performed in triplicates.

In order to ascertain whether the same holds true in THP-1 cell-derived intracellular amastigotes, the first step was to determine the ideal concentration of pentamidine required to suppress ADR in intracellular amastigotes. As seen in S3 Fig, a dose-response analysis revealed that 100 μM of pentamidine inhibited the expression of Ld*AAP3* mRNA. Treatment with 100 μM pentamidine and 500 μM canavanine led to a decrease of both *LdAAP3* and *LdPT* mRNA levels in intracellular amastigotes, while 1 mM NMLAA did not have any significant effect on their expression (Fig 9A, 9B and 9C). Additionally, 100 μM pentamidine, 500 μM canavanine and 1 mM NMLAA did not significantly affect the infectivity of *L*. *donovani* in THP-1 cells (S2D Fig). In order to verify if ADR inhibition by arginine analogues in intracellular amastigotes was not due to changes in cell viability, THP-1 cells were treated with different concentrations of pentamidine, canavanine or NMLAA for 24 h and 48 h, following which they were subjected to MTT assay in order to determine their viability. Cells treated with 100 μM pentamidine, and 1 mM canavanine or NMLAA exhibited 80–100% viability, which was the concentration used for treating infected THP-1 cells for ADR inhibition (S4A–S4C Fig). In conclusion, the above results confirmed that the amidino group is the ligand that binds the arginine sensor. This means that pentamidine and canavanine are recognized by the sensor as “arginine”.

## Discussion

In this study, we have identified that the amidino group on the arginine side chain is the specific ligand that binds the *L*. *donovani* surface arginine sensor, and thereby activates an arginine deprivation response (ADR). We also show that arginine transporter and sensor binding sites are distinct in both axenic and intracellular *L*. *donovani*. Our analysis has indicated that the sensor is more selective in terms of its ligand as compared to the transporter. The arginine sensor not only detects the lack of arginine in the environment but also responds to excessive extracellular arginine by inducing rapid degradation of the LdAAP3 protein and mRNA. This study provides the first identification in *Leishmania* of an intermolecular region or functional group of arginine that interacts with a receptor. In all other sensors identified to date, such intermolecular recognition has not yet been described.

In our present study, it was observed that the arginine structural analogue canavanine and diamidine pentamidine inhibit not only arginine transport but also ADR, in axenic promastigotes as well as intracellular amastigotes. Arginine is a positively charged amino acid with an amidino group (pKa = 13.8) at the distal cap of its side chain [30]. Pentamidine, a diamidine, and a potent anti-protozoal agent, possesses two positively charged amidino moieties which are a part of the guanidium group and have a pKa of 12.1 [28,31,32,33]. Canavanine, on the other hand, is a structural analogue of arginine that has a deprotonated guanidinooxy group (pKa = 6.6) [25,34]. Considering that there is no similarity in the backbone structure of pentamidine and arginine other than the amidino moiety, each of these amidino moieties is the minimal structure which is required to be recognized by the arginine sensor. Any modification of the amidino group, such as the replacement of hydrogen with methyl-group (in case of NMLAA) or replacement of hydrogen with any other groups as in the case of the amidino-modified arginine side-chain analogues and compounds, resulted in their non-recognition by the arginine sensor even when these compounds have an arginine backbone. Among the nine compounds tested in the present study, only pentamidine, canavanine and NMLAA inhibited arginine transport in *Leishmania*, while the others are known to be poor transport inhibitors [8,24]. This provides conclusive evidence that the arginine sensor of *L*. *donovani* is highly specific and exclusive to the amidino group of arginine and also implies that arginine sensing and transport binding sites are distinct in *Leishmania* parasites, in axenic promastigotes and intracellular amastigotes. However, the arginine transporter site is still permissive to change (unlike the sensor site) which does not affect the net charge of the transported compound. This finding opens up the possibility of employing new arginine analogues containing the amidino group as therapeutic agents in leishmaniasis. D-Arginine is an enantiomer of L-Arginine. The parasite arginine sensor seems to have two levels of ligand recognition: i) the enantiomer (D/L) of the primary amine of arginine and ii) the amidino group. Thus, the presence of D-Arginine may not activate ADR even though it has an amidino group. When the chemical ligand lacks primary amine as in the case of pentamidine, the arginine sensor recognizes the presence of amidino group and hence triggers ADR.

The up-regulation of various members of the ADR pathway including LdAAP3, pteridine transporter, folate/biopterin transporter among others upon arginine starvation of *L*. *donovani* suggests that the ADR pathway not only regulates the expression of LdAAP3 but also other transporters of essential compounds such as vitamin B9 (folate) and pteridine which are essential for *Leishmania*. The variation in the RNA degradation profile seen during arginine sufficiency suggests that *LdAAP3* and other members of the ADR pathway including *LdPT* are regulated differently. The mRNA degradation of *LdAAP3* was slower as compared to *LdPT* as it is an central gene in the ADR pathway.

Macrophage phagolysosomes evolve from late endocytic compartments [35] and are the sites of *Leishmania* amastigote differentiation [36]. The arginine concentration in phagolysosomes was found to be 140 μM (Fischer-Weinberger et al, in preparation). We also found that ADR activation in intracellular *L*. *donovani* amastigotes occurred between 24 and 48 h post-infection under physiological levels of arginine (~100 μM), which is considerably higher than the concentration which induces ADR in axenic parasites (5 μM). Thus, intracellular arginine starvation builds up between 24 and 48 h post-infection, which may be the time taken for the depletion of arginine levels from 140 μM (the physiological concentration in phagolysosomes) to 5 μM (which is sufficient for ADR activation in axenic *L*. *donovani*). Hence, it is noteworthy that the axenic *Leishmania* parasite model system established by various research groups including ours [37] is well-suited for deciphering molecular mechanisms in *Leishmania* as this is a clean system without host-protein interference.

Our present study is the first to elucidate the specificity of the parasite arginine sensor, as well as its arginine sufficiency and deficiency responses, both in axenic and intracellular parasites. Sensing nutrient availability in vector or host environment may be essential for parasite survival and growth. Thus, nutrient sensing and transport pathways can be promising drug targets in the protozoan parasite *Leishmania* [38]. Our study also provides evidence that the *L*. *donovani* arginine sensor has a dual function of response to not just arginine deprivation as we have reported earlier [7], but also to arginine sufficiency, and thus maintains homeostasis. Transceptors are dual function solute transporters that concomitantly sense and translocate their substrates, and localize to cell surface and organelle membranes [39]. A well-characterized example of the transceptor is the SSY1 transceptor in *Saccharomyces cerevisiae* [40]. It is part of a membrane-sensing system that detects extracellular amino acids by binding to it [41,42] and signal transmission for detecting the presence of extracellular amino acids is initiated at the cell membrane. Mammals possess the System A amino acid transporter 2 (SNAT2), which also acts as a transceptor that signals and senses neutral amino acid availability [43]. More recently, an arginine trans-membrane sensor (*SLC38A9*) has been identified on the membrane of mammalian lysosomes [44,45]. However, signaling is initiated at the cell membrane where the arginine sensor is most likely localized. Whether the arginine sensor and transporter sites in *L*. *donovani* are located in the same protein or different proteins remains an open question. Also, the existence of multiple arginine transporters, sensors or transceptors implies that arginine is evolutionarily conserved and indispensable in both lower and higher organisms. In recent years, research on flagella-localized transporters in *Leishmania* suggest that these transporters may be involved in ligand sensing. Some of the well-known transporters include glucose transporter 1 (GT1) from *L*. *mexicana* [46] and aquaglyceroporin (AQP1) from *L*. *major* involved in osmoregulation [47]. The phenomenon of sensors localization on the flagella has recently been discussed [48]. In light of this, the fact that arginine is an essential amino acid in *Leishmania*, and semi-essential in mammals, represents its global role in various cellular processes.

In a commentary to our previous paper on the discovery of ADR, McConville [49] suggested that the arginine sensing phenomenon is a metabolic crosstalk between *Leishmania* and the macrophage host. Our finding in this work that ADR is activated in parasites inside phagolysosomes, early in infection, strongly supports this idea. Furthermore, the identification of the arginine intermolecular ligand is unprecedented and provides an efficient tool to further explore host-parasite interaction.

## Supporting information

S1 Fig Changes in cell viability or the presence of extracellular parasites do not affect ADR activation in intracellular amastigotes. A. Cell viability assay of infected THP-1 cells in RPMI medium containing different concentrations of arginine. THP-1 cells, either uninfected or infected with *L*. *donovani* in RPMI medium containing 0, 0.1 mM, 0.5 mM or 1.5 mM arginine for 48 h were incubated with diluted MTT solution for 2 h. Thereafter, stopping solution consisting of isopropanol containing 5% formic acid was added to the cells, and they were incubated for 20 min. The absorbance was then measured at 570 nm, and the percentage cell viability was calculated. The results are representative of three independent experiments performed in triplicates. B. Real-time PCR analysis of *L*. *donovani* promastigotes cultured in different concentrations of arginine. *L*. *donovani* promastigotes were cultured in RPMI medium containing 0.1 mM or 0.5 mM arginine for 48 h. The total RNA was extracted, and the resulting cDNA was analyzed by real-time PCR using primers specific for *LdAAP3* and *LdPT*. The results are expressed as fold-change of control (2 h infected cells). Values are mean ± S.E.M. (n = 3). The results are representative of three independent experiments performed in triplicates. C. Cell viability assay of *L*. *donovani* promastigotes cultured in medium containing different arginine concentrations. *L*. *donovani* promastigotes were cultured in RPMI medium containing 0.1 mM, 0.5 mM or 1.5 mM arginine for 48 h. They were then incubated with diluted MTT solution for 3 h. Stop solution comprising of isopropanol and 20% SDS in a 1:1 ratio was added to the cells for 30 min., followed by measurement of absorption at 570 nm and calculation of the percentage cell viability. The results are representative of three independent experiments performed in triplicates.
(TIF)

S2 Fig Infectivity of *L*. *donovani* in THP-1 cells. A. THP-1 cells were infected with *L*. *donovani* in RPMI medium containing 0, 0.1 mM, 0.5 mM or 1.5 mM arginine for 48 h. They were then stained with Giemsa and the number of infected cells were counted visually. B. THP-1 cells were infected with *L*. *donovani* in RPMI medium containing 0.1 mM arginine for 2 h, 24 h and 48 h. They were then stained with Giemsa and the number of infected cells were counted visually. C. THP-1 cells were infected with *L*. *donovani* in RPMI medium containing 0.1 mM arginine for 48 h. They were then treated with 1 mM or 5 mM of arginine for 2 h, stained with Giemsa and the number of infected cells were counted visually. D. THP-1 cells infected with *L*. *donovani* in RPMI medium containing 0.1 mM arginine for 48 h were treated with 100 μM pentamidine (A), 500 μM canavanine (B), or 1 mM NMLAA (C) for 2 h. Infected and untreated cells were used as control. The cells were then stained with Giemsa and the number of infected cells were counted visually. The results are representative of three independent experiments.
(TIF)

S3 Fig The minimum concentration of pentamidine required for ADR inhibition in intracellular amastigotes. THP-1 cells infected with *L*. *donovani* in RPMI medium containing 0.1 mM arginine for 48 h were treated with 0, 5 μM, 20 μM, 37.6 μM, 50 μM and 100 μM pentamidine for 2 h. The total RNA was extracted, and the resulting cDNA was subjected to real-time PCR analysis using primers specific for *LdAAP3*. The results are expressed as fold-change of control (2 h infected and untreated cells). Values are mean ± S.E.M. (n = 3). The results are representative of three independent experiments performed in triplicates.
(TIF)

S4 Fig Changes in cell viability do not affect the inhibition of ADR by arginine analogues in intracellular amastigotes. A-C. THP-1 cells treated with 0, 0.1 mM, 0.5 mM or 1 mM pentamidine, or with 0, 1 mM, 2.5 mM, 5 mM or 10 mM of canavanine or NMLAA for 24 h and 48 h were incubated with diluted MTT solution for 2 h. Thereafter, stopping solution consisting of isopropanol containing 5% formic acid was added to the cells, and they were incubated for 20 min. The absorbance was then measured at 570 nm, and the percentage cell viability was calculated. The results are representative of three independent experiments performed in triplicates.
(TIF)
