# Supplementary figures and images for: The arginine sensing and transport binding sites are distinct in the human pathogen Leishmania
Source: PLoS Negl Trop Dis. 2019 Apr 24;13(4):e0007304. doi: 10.1371/journal.pntd.0007304 (PMC6502434; doi:10.1371/journal.pntd.0007304)

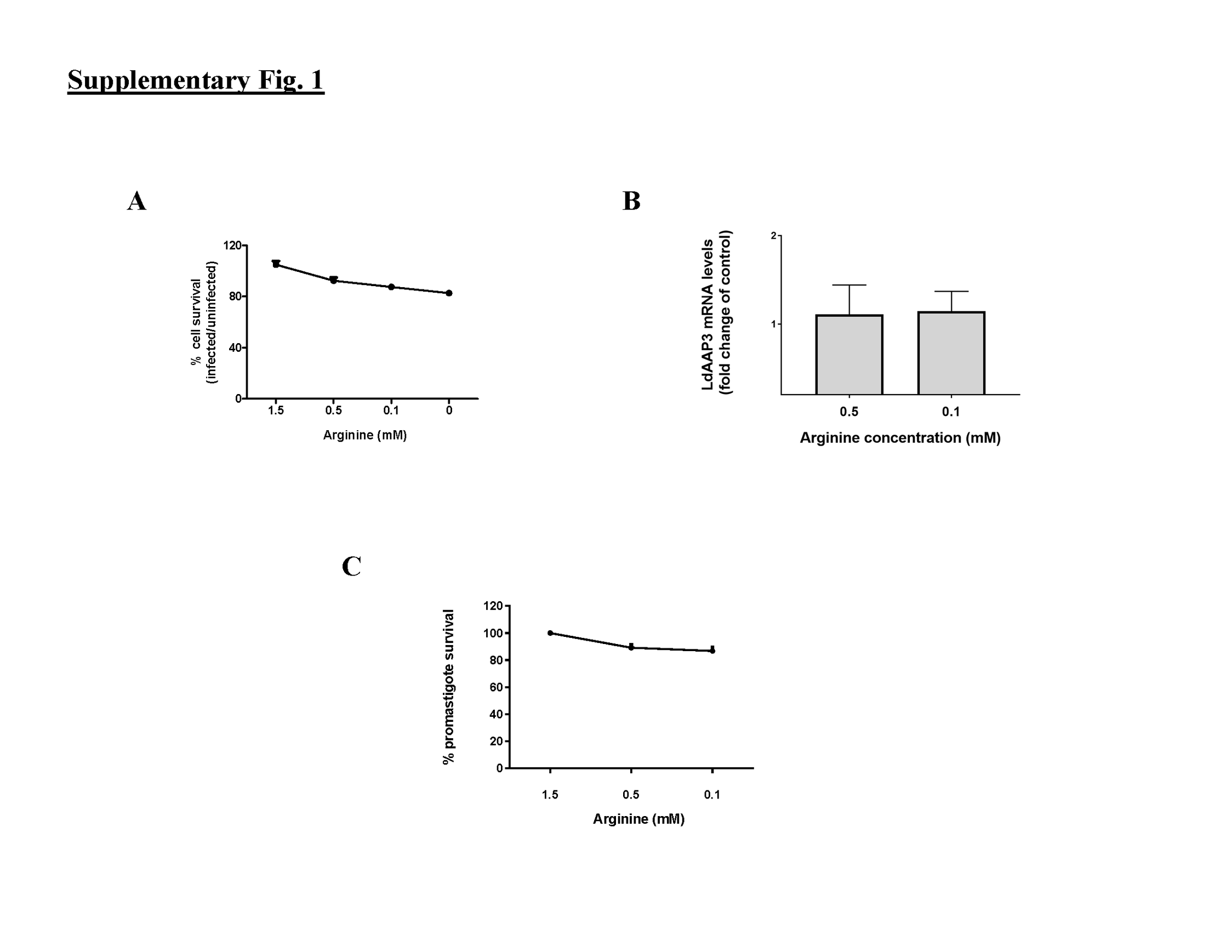

Supplement: S1 Fig — A. Cell viability assay of infected THP-1 cells in RPMI medium containing different concentrations of arginine. THP-1 cells, either uninfected or infected with L. donovani in RPMI medium containing 0, 0.1 mM, 0.5 mM or 1.5 mM arginine for 48 h were incubated with diluted MTT solution for 2 h. Thereafter, stopping solution consisting of isopropanol containing 5% formic acid was added to the cells, and they were incubated for 20 min. The absorbance was then measured at 570 nm, and the percentage cell viability was calculated. The results are representative of three independent experiments performed in triplicates. B. Real-time PCR analysis of L. donovani promastigotes cultured in different concentrations of arginine. L. donovani promastigotes were cultured in RPMI medium containing 0.1 mM or 0.5 mM arginine for 48 h. The total RNA was extracted, and the resulting cDNA was analyzed by real-time PCR using primers specific for LdAAP3 and LdPT. The results are expressed as fold-change of control (2 h infected cells). Values are mean ± S.E.M. (n = 3). The results are representative of three independent experiments performed in triplicates. C. Cell viability assay of L. donovani promastigotes cultured in medium containing different arginine concentrations. L. donovani promastigotes were cultured in RPMI medium containing 0.1 mM, 0.5 mM or 1.5 mM arginine for 48 h. They were then incubated with diluted MTT solution for 3 h. Stop solution comprising of isopropanol and 20% SDS in a 1:1 ratio was added to the cells for 30 min., followed by measurement of absorption at 570 nm and calculation of the percentage cell viability. The results are representative of three independent experiments performed in triplicates. (TIF) [file pntd.0007304.s001.tif]

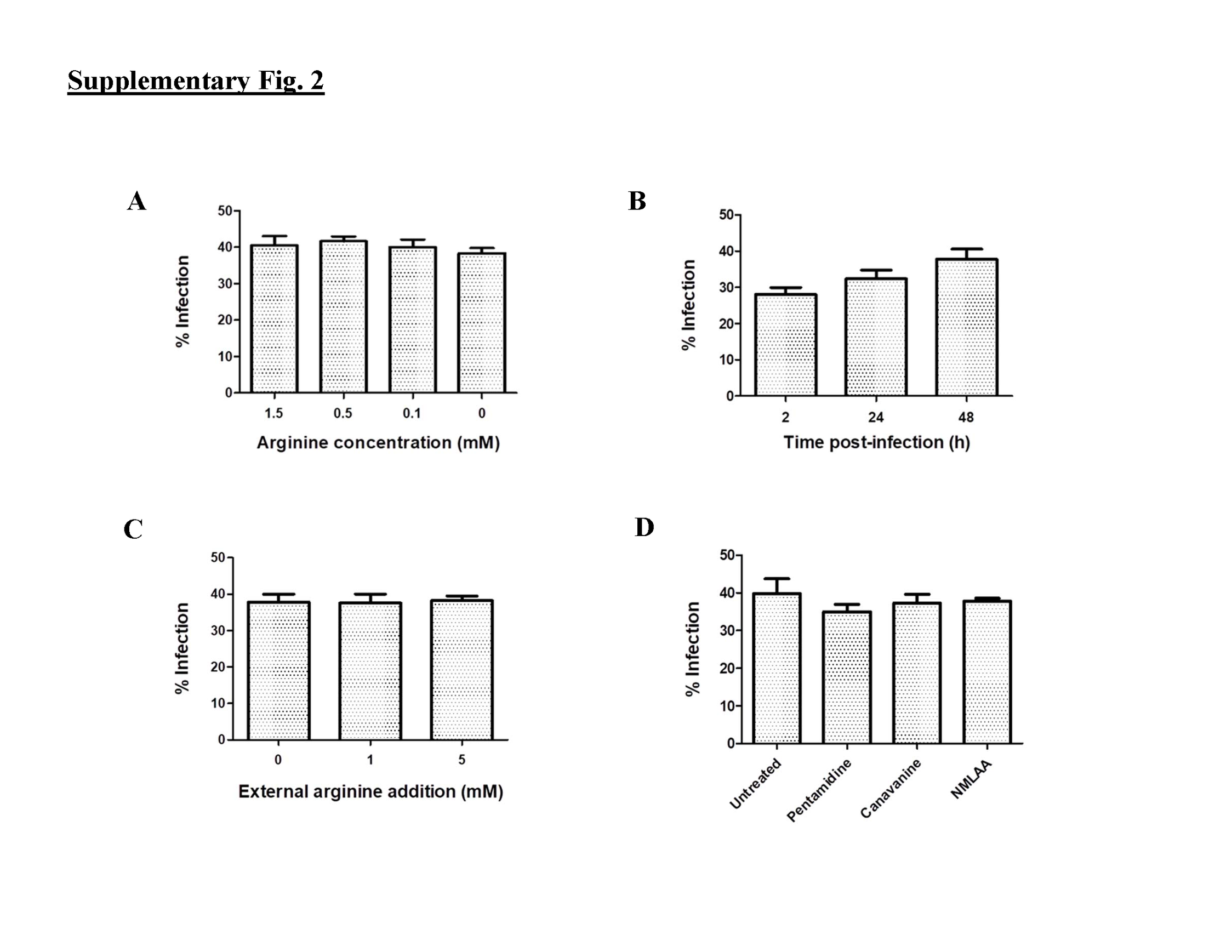

Supplement: S2 Fig — A. THP-1 cells were infected with L. donovani in RPMI medium containing 0, 0.1 mM, 0.5 mM or 1.5 mM arginine for 48 h. They were then stained with Giemsa and the number of infected cells were counted visually. B. THP-1 cells were infected with L. donovani in RPMI medium containing 0.1 mM arginine for 2 h, 24 h and 48 h. They were then stained with Giemsa and the number of infected cells were counted visually. C. THP-1 cells were infected with L. donovani in RPMI medium containing 0.1 mM arginine for 48 h. They were then treated with 1 mM or 5 mM of arginine for 2 h, stained with Giemsa and the number of infected cells were counted visually. D. THP-1 cells infected with L. donovani in RPMI medium containing 0.1 mM arginine for 48 h were treated with 100 μM pentamidine (A), 500 μM canavanine (B), or 1 mM NMLAA (C) for 2 h. Infected and untreated cells were used as control. The cells were then stained with Giemsa and the number of infected cells were counted visually. The results are representative of three independent experiments. (TIF) [file pntd.0007304.s002.tif]

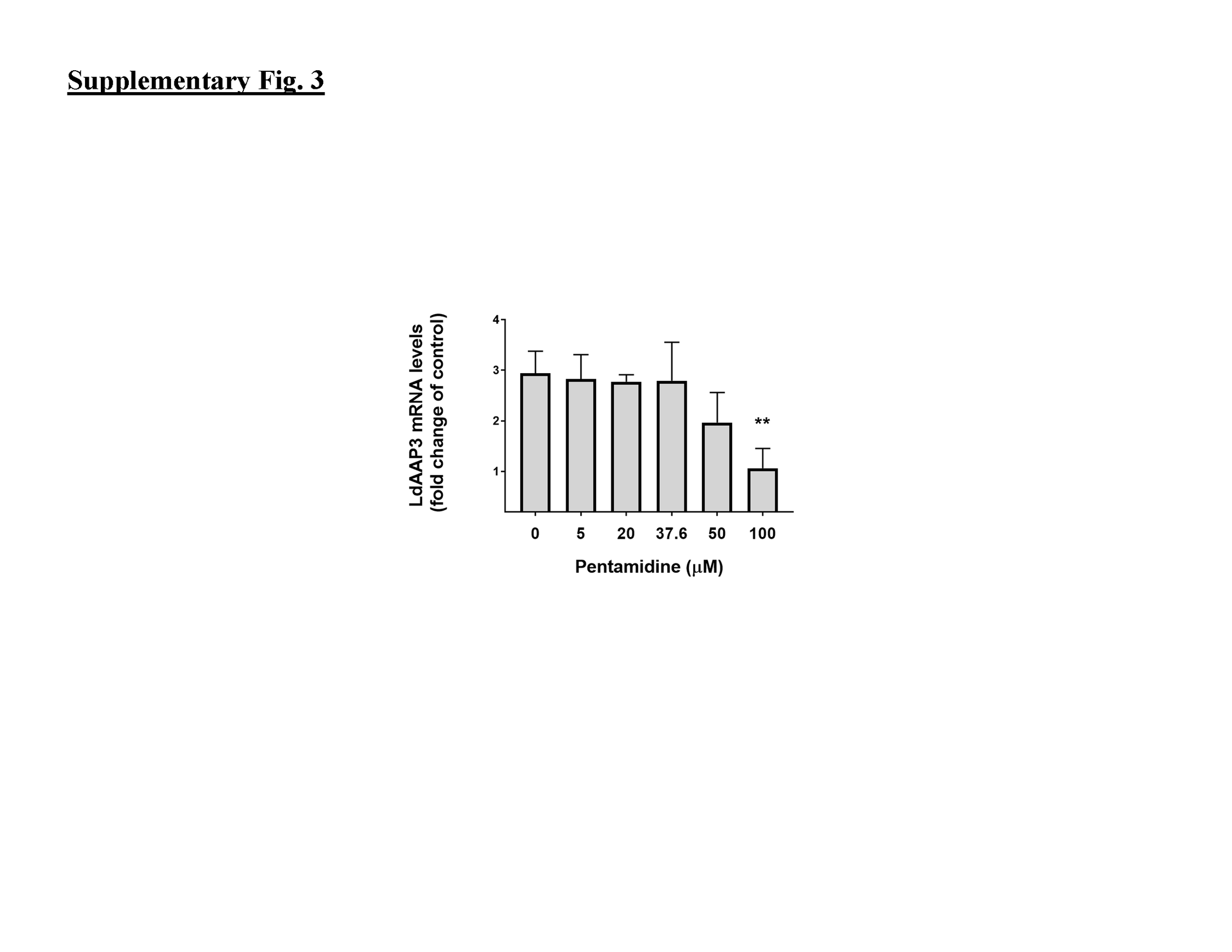

Supplement: S3 Fig — THP-1 cells infected with L. donovani in RPMI medium containing 0.1 mM arginine for 48 h were treated with 0, 5 μM, 20 μM, 37.6 μM, 50 μM and 100 μM pentamidine for 2 h. The total RNA was extracted, and the resulting cDNA was subjected to real-time PCR analysis using primers specific for LdAAP3. The results are expressed as fold-change of control (2 h infected and untreated cells). Values are mean ± S.E.M. (n = 3). The results are representative of three independent experiments performed in triplicates. (TIF) [file pntd.0007304.s003.tif]

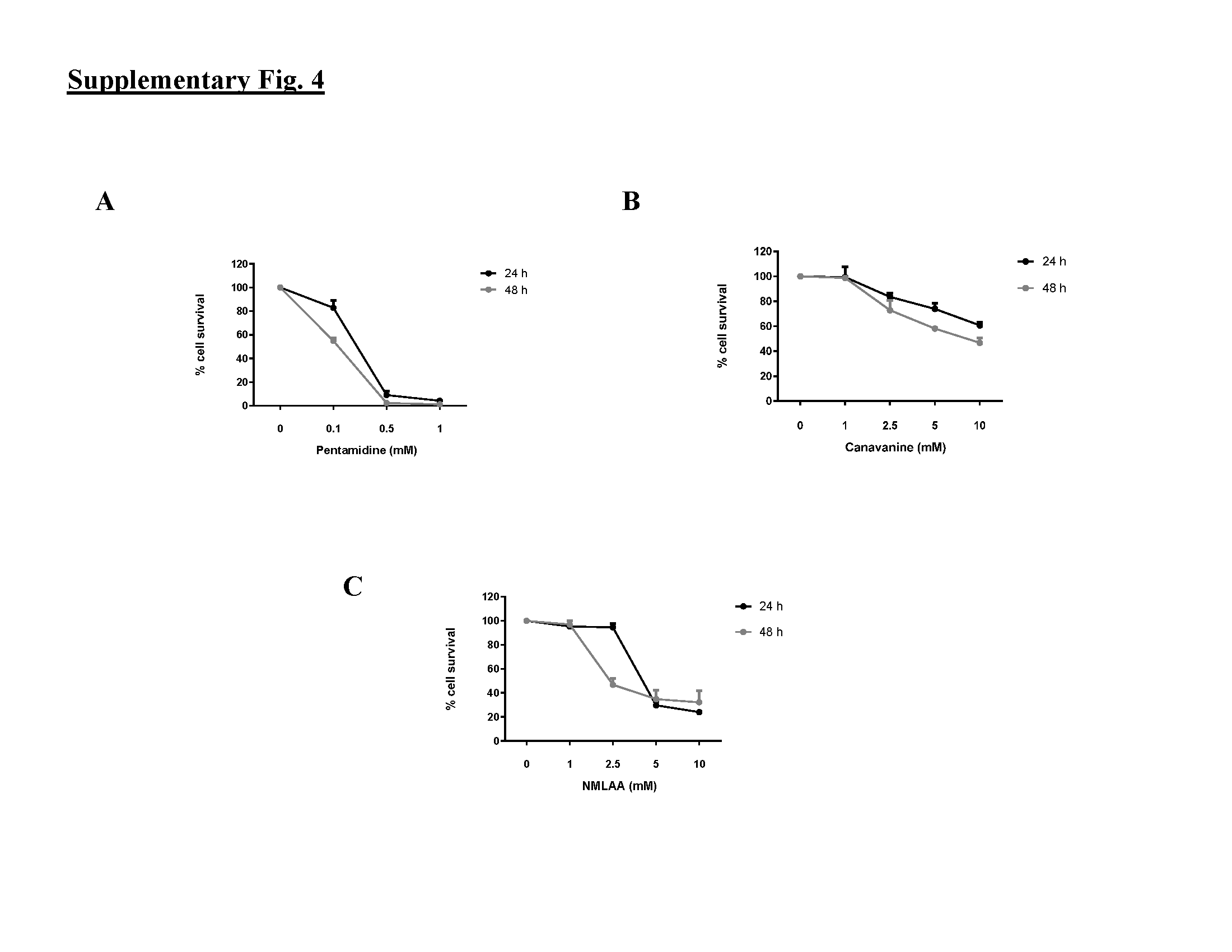

Supplement: S4 Fig — A-C. THP-1 cells treated with 0, 0.1 mM, 0.5 mM or 1 mM pentamidine, or with 0, 1 mM, 2.5 mM, 5 mM or 10 mM of canavanine or NMLAA for 24 h and 48 h were incubated with diluted MTT solution for 2 h. Thereafter, stopping solution consisting of isopropanol containing 5% formic acid was added to the cells, and they were incubated for 20 min. The absorbance was then measured at 570 nm, and the percentage cell viability was calculated. The results are representative of three independent experiments performed in triplicates. (TIF) [file pntd.0007304.s004.tif]
